# A Gaze Estimation Method Based on Spatial and Channel Reconstructed ResNet Combined with Multi-Clue Fusion

**DOI:** 10.3390/jimaging11040099

**Published:** 2025-03-27

**Authors:** Zhaoyu Shou, Yanjun Lin, Jianwen Mo, Ziyong Wu

**Affiliations:** 1 School of Information and Communication, Guilin University of Electronic Technology, Guilin 541004, China; guilinshou@guet.edu.cn (Z.S.); 22022201046@mails.guet.edu.cn (Y.L.); jwmo@guet.edu.cn (J.M.); 2 Guangxi Wireless Broadband Communication and Signal Processing Key Laboratory, Guilin University of Electronic Technology, Guilin 541004, China; 3 Guangxi Key Laboratory of Trusted Software, Guilin University of Electronic Technology, Guilin 541004, China

**Keywords:** gaze estimation, SCConv, ResNet

## Abstract

The complexity of various factors influencing online learning makes it difficult to characterize learning concentration, while Accurately estimating students’ gaze points during learning video sessions represents a critical scientific challenge in assessing and enhancing the attentiveness of online learners. However, current appearance-based gaze estimation models lack a focus on extracting essential features and fail to effectively model the spatio-temporal relationships among the head, face, and eye regions, which limits their ability to achieve lower angular errors. This paper proposes an appearance-based gaze estimation model (RSP-MCGaze). The model constructs a feature extraction backbone network for gaze estimation (ResNetSC) by integrating ResNet and SCConv; this integration enhances the model’s ability to extract important features while reducing spatial and channel redundancy. Based on the ResNetSC backbone, the method for video gaze estimation was further optimized by jointly locating the head, eyes, and face. The experimental results demonstrate that our model achieves significantly higher performance compared to existing baseline models on public datasets, thereby fully confirming the superiority of our method in the gaze estimation task. The model achieves a detection error of 9.86 on the Gaze360 dataset and a detection error of 7.11 on the detectable face subset of Gaze360.

## 1. Introduction

Gaze estimation is a challenging research task that has emerged in recent years. It is influenced by variations in head pose, facial attributes, environmental lighting, and other factors. Currently, gaze estimation in human–computer interaction is primarily applied in scenarios such as unmanned stores, driver fatigue detection, and virtual reality (VR). Online education, as an emerging form of human–computer interaction, also holds significant research value for gaze estimation in the process where students watch teaching videos or live broadcasts. To better evaluate students’ learning concentration, learning ability, and learning outcomes, accurately capturing the students’ gaze direction has become key to providing feedback in teaching. Compared with gaze estimation in static images, gaze estimation in video scenarios involves more complex spatiotemporal variations. The dynamic changes in the eyes, head, and face, as well as their intrinsic connections, are crucial for accurately characterizing the direction of gaze. Despite the significant efforts made in the field of video gaze estimation as reported in the literature [1,2,3,4,5], a deep correlation mechanism among the clues of the eyes, head, and face has not yet been established. Guan et al. [6] proposed an end-to-end video gaze estimation model by capturing the spatiotemporal interaction context of the head–face–eye, effectively realizing the information interaction between the global descriptive cues of the head and face and the local fine-grained cues of the eyes. However, using gaze estimation as the primary criterion for assessing students’ learning concentration in online classrooms imposes higher requirements on feature extraction and redundancy filtering. Therefore, gaze estimation models for online classroom scenarios should focus on extracting key features from the face and effectively filtering out redundant information in order to more accurately predict the students’ gaze direction and precisely characterize their learning concentration. However, in the existing appearance-based gaze estimation research, there is a lack of attention to key facial features and the removal of redundant information.

To address the above issues, a ResNet network with joint spatial information reconstruction and a multi-cue gaze estimation method called RSP-MCGaze are proposed. This method is a gaze estimation approach based on videos of online learning. The backbone network of RSP-MCGaze replaces the 3 × 3 convolution in ResNet with Spatial-Channel Convolution (SCConv). This modification enhances the backbone network’s ability to extract key features (head, eyes, and face) from video segments and removes redundant spatial and channel information. Doing so reduces the interference from irrelevant factors in the subsequent gaze estimation cue fusion and decreases computational costs. A new backbone network, ResNetSC, is designed for the gaze estimation task in online classrooms based on ResNet and SCConv. Overall, the main contributions of this study are summarized as follows:

1. A gaze estimation feature extraction backbone network, ResNetSC, combining ResNet and SCConv, is proposed. By replacing the traditional 3 × 3 convolution with SCConv, the network not only enhances the model’s ability to extract important features but also significantly reduces spatial and channel redundancy, thereby decreasing the number of model parameters.
2. The ResNetSC backbone network is combined with the joint localization of the head, eyes, and face to jointly optimize the video gaze estimation model. A new gaze estimation method for online learning is proposed, which improves performance and accuracy.

The remainder of this paper is organized as follows: Section 2 introduces the related work. Section 3 presents the gaze estimation model based on spatial-channel reconstruction and multi-cue localization. Section 4 describes the experimental process, analyses the experimental results, and evaluates the model’s performance. The final section concludes the paper and provides future work.

## 2. Related Work

Gaze estimation mainly includes three categories of methods: 2D eye feature regression methods, 3D eye model recovery methods, and appearance-based methods. The 2D eye feature regression methods and 3D eye model recovery methods rely on geometric features such as contours, reflections, and eye structures. With the help of infrared cameras or other specialized devices, these two methods can improve the accuracy of geometric feature extraction. In contrast, appearance-based gaze estimation methods directly learn the mapping function from images to gaze directions. Unlike the 2D eye feature regression and 3D eye model recovery methods, appearance-based methods do not require specialized equipment to detect geometric features. Instead, they rely solely on image pixels and deep features to estimate gaze directions, significantly simplifying the complexity of gaze estimation tasks. Currently, researchers widely employ methods such as neural networks, Gaussian processes, convolutional neural networks, and adaptive linear regression. However, when confronted with complex facial appearance images, appearance-based methods still face many challenges. To address the impact of varying lighting conditions on eye images in different environments, Huang et al. [7] proposed a variational inference model, namely the Variational Gaze Estimation Network (VGE-Net). This model generates multiple gaze maps as complementary candidates, which are supervised by the ground-truth gaze map. Additionally, it employs a simple attention mechanism to adaptively fuse the predicted gaze directions on the candidate gaze maps using a regression network. The regression of gaze direction using a single-eye image is rather complex and inaccurate. To address this issue, Cheng et al. [1] proposed the FAR-Net algorithm, which employs an asymmetric approach to estimate the three-dimensional gaze angles of both eyes. This method assigns asymmetric weights to the loss of each eye and then sums up these losses. The model demonstrates excellent performance on several public datasets. To address the insufficient generalization ability of existing gaze estimation methods, Xu et al. [8] proposed a new region generalization method based on gaze-consistent features. By treating factors unrelated to gaze as adverse disturbances and introducing these disturbances into the training data, the model is prevented from fitting to these gaze-unrelated factors and can only fit features consistent with gaze. To address the impact of unobservable head poses on gaze estimation, Hisadome et al. [9] proposed a generalizable multi-view gaze estimation task and a cross-view feature fusion method, which addresses the limited generalization performance for unseen head poses. To address the generalizability of gaze estimation across domains, Yin et al. [10] proposed a new framework called CLIP-Gaze. This framework leverages a pre-trained visual-language model to utilize its transferable knowledge and is the first to employ a cross-modal approach combining vision and language for the gaze estimation task. To explore the correlation and interaction between the face and eyes in the gaze estimation task, Cheng et al. [4] proposed a coarse-to-fine adaptive network (CA-Net). This network first uses a face image to predict the main gaze angle and adapts it to the residual estimated from the eye crop. Subsequently, a dual-graph model was proposed to bridge the main gaze and the residual estimated from the eye crop. Transformers equipped with self-attention modules have brought high performance to various computer vision tasks. Jun et al. [11] used a Transformer with a self-attention module to extract key gaze features from high-variance images. Convolutional projections were employed to effectively filter out inattentive gaze information, while deconvolutional layers were used to preserve image detail features. Similarly, AGE-Net proposed two parallel networks for each eye image: one using a CNN to generate feature vectors and the other employing an attention-based network to generate weighted feature vectors [12]. Li et al. [13] proposed the Static Transformer Module (STM), which uses a multi-head self-attention mechanism to fuse fine-grained eye features and coarse-grained face features. They also introduced an innovative Recurrent Neural Network (RNN) unit, namely the Temporal Difference Module (TDM), which can be used to extract dynamic features. Finally, the STM and TDM are integrated into the Static Transformer through the Temporal Difference Network (STTDN). Wu et al. [14] proposed an appearance-based end-to-end learning network architecture and introduced an attention mechanism called the Efficient Gaze Network (EG-Net). This network employs a two-branch structure for gaze estimation: a base CNN is used for the full-face image, while an Efficient Eye Network (EE-Net), which is upscaled from the base CNN, is used for the left and right eye images. The EE-Net uses a set of constant coefficients to extract eye features, uniformly scaling the depth, width, and resolution of the base CNN. It also adaptively weights the left and right eye images through an attention network based on their “image quality”. Zhang et al. [15] developed the large-scale, high-resolution ETH-XGaze dataset under constraints of extreme head poses and gaze variations. Zhang et al. [16] proposed a method for estimating the gaze direction of multiple people, which starts from a large input image. However, this method cannot predict the origin of the gaze and, therefore, cannot directly compute the Point of Gaze (PoG). To address this issue, Haldun Balim et al. [17] proposed an end-to-end learning method called EFE, which can start from camera frames and end with the Point of Gaze (PoG), thus perfectly achieving end-to-end training. These studies have propelled the continuous advancement of gaze estimation technology, providing diverse technical pathways to enhance estimation accuracy and generalization ability.

In the traditional ResNet network, the classic 3 × 3 convolution accounts for a large number of model parameters and FLOPs. Existing studies [18,19,20,21] are all committed to reducing redundancy in deep neural networks and have confirmed that redundancy is not only present in dense models but is also widely distributed across the spatial and channel dimensions of feature maps. However, these studies often focus on reducing redundancy in a single dimension, making it difficult to comprehensively address the issue of feature redundancy. To tackle this problem, Li et al. [22] proposed a novel CNN compression method that can simultaneously reduce redundancy in both spatial and channel dimensions, known as SCConv.

## 3. Methodology

**Figure 1:**
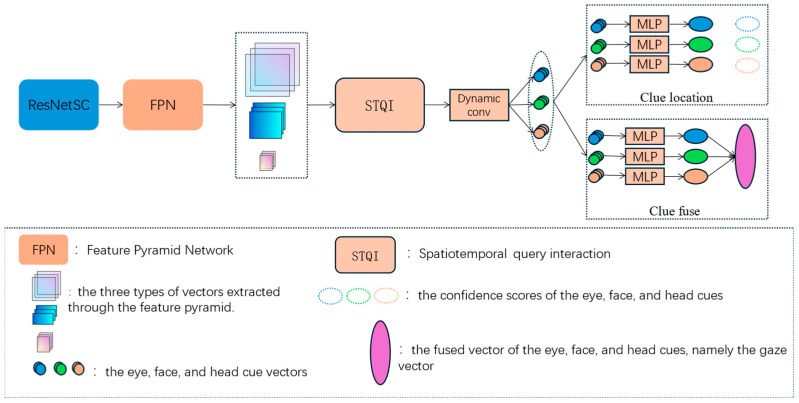
Overall Framework of RSP-MCGaze.

The overall framework of RSP-MCGaze consists of a backbone network, spatiotemporal query interaction, localization heads, and gaze fusion heads. The backbone network is the ResNet network optimized by spatial-channel reconstruction, namely ResNetSC. Finally, gaze estimation is accomplished by jointly considering the spatiotemporal interactions among the head, eyes, and face, using dynamic convolution. The overall block diagram is shown in Figure 1.

### 3.1. Spatial-Channel Reconstruction Convolution

Applying the Spatial Reconstruction Unit (SRU) to the convolutional layers of ResNet not only separates spatially rich features from those with little or no spatial information but also reconstructs the features. Therefore, integrating SRU into the ResNet network as the backbone can not only reduce redundancy in spatial feature information but also enhance their correlation.

**Figure 2:**
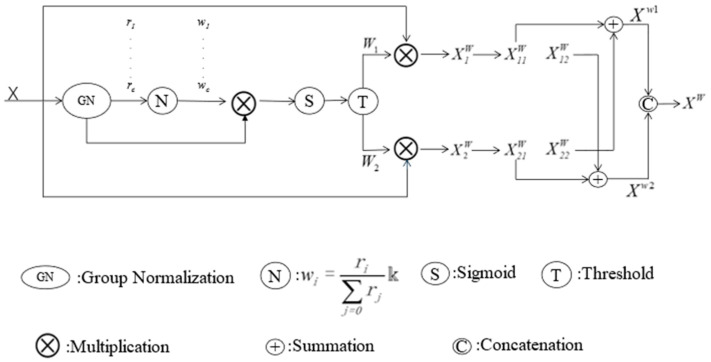
The architecture of the Spatial Reconstruction Unit.

The spatial Reconstruction Unit (SRU) consists of two parts: separation and reconstruction. The main steps are illustrated in Figure 2. The separation primarily refers to distinguishing feature maps with abundant spatial information from those with less spatial information (redundant). In the separation part, SRU assesses the amount of spatial information carried by the feature maps through the scaling factors in the Group Normalization (GN) layer and ultimately divides the feature maps into two parts, as shown in Equation (1):

(1) $$\begin{array}{l} X_{1}^{w}=W_{1}⊗X\\ X_{2}^{w}=W_{2}⊗X \end{array}$$

where $X_{1}^{w}$ is informative and representative of spatial features, $X_{2}^{w}$ represent the features that lack or contain very little information, which are considered redundant; *W*_1_ represents the weight of information above the threshold, while *W*_2_ denotes the weight of non-information below the threshold and ⊗ is element-wise multiplication.

The second part of the Spatial Reconstruction Unit (SRU): The reconstruction part employs a cross-reconstruction operation to fully combine the two distinct weighted information features, thereby strengthening the information flow between them:

(2) $$\begin{array}{l} X_{11}^{w}⊕X_{22}^{w}=X^{w1}\\ X_{21}^{w}⊕X_{12}^{w}=X^{w2}\\ X^{w1}\cup X^{w2}=X^{w} \end{array}$$

where ⊕ is element-wise summation, ∪ is concatenation, $X^{w1}$ and $X^{w2}$ are the features obtained through cross-reconstruction, *X_w_* represents the spatially reconstructed feature output by the SRU at the final stage.

**Figure 3:**
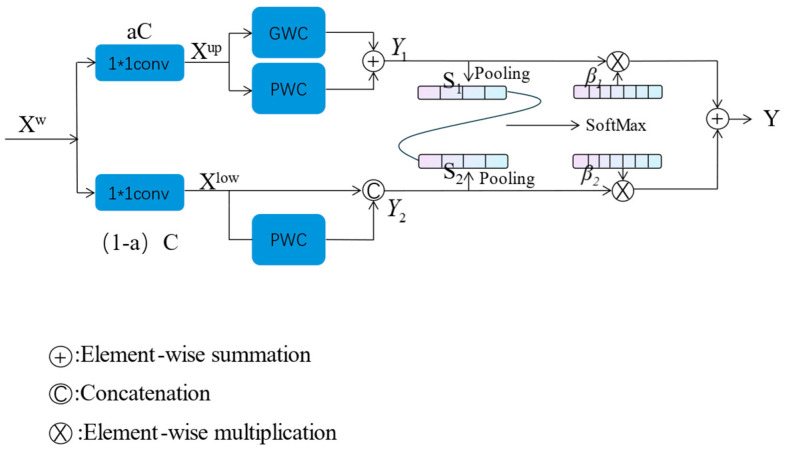
The architecture of the Channel Reconstruction Unit.

Channel Reconstruction Unit (CRU) utilizes a split-transform-fuse strategy. In the ResNet network, repeated 3 × 3 convolutions are typically used for feature extraction, which can lead to redundant feature mappings in the channel dimension. CRU can replace the standard k × k convolution to reduce channel redundancy through split-transform-fuse, as shown in Figure 3:

Splitting: The refined spatial features obtained through SRU are split into two parts: *aC* channels and (*1 − a*)*C* channels. Then, 1 × 1 convolutions are used to compress the channels of the feature maps in each part, thereby improving computational efficiency.

Transformation: After splitting, two parts of features, *X_up_* and *X_low_*, are obtained. The upper-level transformation employs efficient convolution operations (Grouped Weighted Convolution, GWC, and Pointwise Weighted Convolution, PWC). Compared to the standard k × k convolution, these methods reduce the number of parameters and computational cost without causing information loss.

(3) $$Y_{1}=M^{G}X_{up}+M^{P_{1}}X_{up}$$

where *M^G^* and *M*^*P*_1_^ are the learnable weight matrices of GWC (Grouped Weighted Convolution) and PWC (Pointwise Weighted Convolution). *X_up_* and *Y*_1_ represent the input and output feature maps of the upper part, respectively.

(4) $$Y_{2}=M^{P_{2}}X_{low}\cup X_{low}$$

where *M*^*P*_2_^ is the learnable weight matrices of PWC, *X_low_* is the input feature map of the lower part, and *Y*_2_ is the output feature map of the lower part.

Fusion: The CRU employs a simplified SKNet to adaptively fuse the transformed features. First, global spatial information is collected using average pooling.

(5) $$S_{m}=Pooling(Y_{m})=\frac{1}{H\times W}\sum_{i=1}^{H}\sum_{j=1}^{W}Y_{c}(i,j),m=1,2$$

Then, the global information descriptors of the upper and lower parts are concatenated to generate a feature importance vector.

(6) $$\begin{array}{l} \beta_{1}=\frac{e^{s_{1}}}{e^{s_{1}}+e^{s_{2}}}\\ \beta_{2}=\frac{e^{s_{2}}}{e^{s_{1}}+e^{s_{2}}}\\ \beta_{1}+\beta_{2}=1 \end{array}$$

where $\beta_{1}$ and $\beta_{2}$ are feature importance vectors.

Finally, under the guidance of the feature importance vector, the upper and lower features are concatenated in the channel direction.

(7) $$Y=\beta_{1}Y_{1}+\beta_{2}Y_{2}$$

where *Y*_1_ is upper-part features, *Y*_2_ is lower-part features, and *Y* is channel-refined features.

### 3.2. Spatiotemporal Query Interaction

In the gaze estimation task designed in this paper, a spatiotemporal interaction query module is used [23], and the query cues from the eye, head, and face parts are combined to form the module. This helps to better locate spatial and temporal hierarchical information.

(8) $$\left\{Y_{f}^{t},Y_{h}^{t},Y_{e}^{t}\}=MHSA(\{Y_{f}^{t},Y_{h}^{t},Y_{e}^{t}\}\right)$$

where $Y_{f}^{t}$ denotes the refined facial features, $Y_{h}^{t}$ represents the refined head features, $Y_{e}^{t}$ corresponds to the refined eye features. By querying the face, head, and eyes and using Multi-Head Self-Attention (MHSA), the interaction between global head and face information and local eye information can be better promoted. This allows the gaze features to capture both global and local spatial features, enhancing the accuracy of gaze estimation. In addition, after spatial interaction, a self-attention mechanism is designed to facilitate temporal information interaction.

(9) $${\left\{Y_{clue}^{t}\right\}}_{t=1}^{T}=MHSA({\left\{Y_{clue}^{t}\right\}}_{t=1}^{T})$$

After spatiotemporal query interaction, dynamic convolution is also incorporated to continuously update the cue features in iterations and ensure a high correlation among the features. The cue features updated by dynamic convolution are finally used for the subsequent gaze estimation task.

### 3.3. Clue Localization Heads and Gaze Fusion Heads

The clue localization head takes a continuously updated query and identifies the region of interest that the query focuses on. Within the clue localization head, a Multilayer Perceptron (MLP) with sigmoid normalization is used to represent the regions of interest in each query cue.

Since there are three query clues—face, head, and eyes—three separate Multilayer Perceptron (MLP) are employed to localize the regions of interest for each of these different parts. Each cue has its own localization head. $s_{clue}\in {\mathbb{R}}^{T}$, clue∈{face,head,eyes}.

(10) $$\begin{array}{l} s_{clue}=Sigmoid(MLP_{clue}^{s}(q_{clue}^{*}))\\ b_{clue}=MLP_{clue}^{b}(Y_{clue}^{*}) \end{array}$$

where *b_clue_* represents the clue localization region, and *s_clue_* denotes different clue heads (including the head, face, and eyes).

In the gaze fusion head, for the updated query features of the three clues, three distinct MLPs are employed to regress the gaze vectors into *g_clue_*, as shown in Equation (11).

(11) $$g_{clue}=MLP_{clue}^{g}(Y_{clue}^{*})$$

Similarly, three Multilayer Perceptrons (MLPs) are utilized to perform confidence processing on the predicted vectors of the three parts, as illustrated in Equation (12).

(12) $$c_{clue}=MLP_{clue}^{c}(Y_{clue}^{*})$$

Finally, a fully connected layer multiplies the three vectors with their corresponding confidence cues to form the fused vector for gaze estimation.

(13) $$g_{f}=FC\{g_{face}*c_{face}+g_{head}*c_{head}+g_{eyes}*c_{eyes}\}$$

where *g_f_* denotes the hybrid vector representing gaze., and *c_clue_* is the confidence level cues of the three parts.

### 3.4. Loss Function

In this study, multiple loss functions are designed to optimize the model. First, we employ the arccos loss to supervise gaze estimation.

(14) $$L_{arccos}=arccos\frac{g\cdot \hat{g}}{\left\|g\right\|\cdot \left\|\hat{g}\right\|}$$

where *g* denotes the ground truth gaze, while $\hat{g}$ represents the predicted gaze output.

In this study, *L_cls_* and *L_box_* are employed to supervise the presence state and bounding box position *b_clue_* of the clue regions. *L_cls_* denotes the focal loss [24], while *L_box_* represents a combination of *L*_1_ loss and GIoU loss for bounding box regression [25]. The loss function is formulated as shown in Equation (15).

(15) $$L_{anchor}=\sum_{t=0}^{T-1}\sum_{clue}\left(L_{box}(b_{clue}^{t},{\hat{b}}_{clue}^{t})+L_{cls}(s_{clue}^{t},{\hat{s}}_{clue}^{t})\right)$$

To minimize the prediction errors between each clue and its corresponding ground truth, this study introduces a dedicated loss function to supervise the gaze prediction outcomes for each individual clue.

(16) $$L_{gaze}=\sum_{t=0}^{T-1}\left(L_{arccos}(g_{f}^{t},{\hat{g}}^{t}\right)+\sum_{clue}L_{arccos}(g_{clue}^{t},{\hat{g}}^{t}))$$

where *clue* ∈ (*head*, *face*, *eye*), *t* denotes the timestamp of the *t*-th frame. The introduction of the temporal frame index *t* in the loss function is to prepare for the subsequent incorporation of temporal regularization.

Then, we incorporate a temporal regularization term to ensure temporal stability, with the loss function formulated as shown in Equation (17).

(17) $$J_{temp}=\sum_{t=1}^{T-2}\left|2\cdot {\hat{g}}_{f}^{t}-{\hat{g}}_{f}^{t-1}-{\hat{g}}_{f}^{t-1}\right|$$

where ${\hat{g}}_{f}^{t}$ denotes the *t*-th frame of the output gaze.

Finally, our overall loss function is designed as

(18) $$L_{total}=L_{anchor}+\lambda_{1}L_{gaze}+\lambda_{2}J_{temp}$$

where $\lambda_{1}$, $\lambda_{2}$ represent the hyperparameters in the loss function. In experiments, they are set to 6 and 1, respectively.

## 4. Experimental Results and Analysis

### 4.1. Datasets

To verify the superiority of RSP-MCGaze, experiments were conducted on the challenging public dataset Gaze360. The dataset includes 238 subjects and contains annotations for 3D gaze, head pose, and imaging distance in both indoor and outdoor environments.

RSP-MCGaze was also tested on the detectable face subset of Gaze360. In Gaze360, some samples fail to capture clear head, eye, and face information, making them unsuitable for training and testing appearance-based gaze estimation methods. Studies have evaluated the detectable face subset of the Gaze360 dataset and obtained a test-worthy subset with a high value [26,27,28].

### 4.2. Experimental Setup

The number of iterations N was set to 4. The AdamW optimizer was employed for training with a batch size of 8 and a learning rate of 1 × 10^−4^. The input image size was 448 × 448. The model was trained for 13,000 iterations, with the learning rate reduced by a factor of 0.1 at 12,000 iterations. When training and evaluating using the entire Gaze360 dataset, following the baseline [29], the input images were resized to 224 × 224, and the batch size was set to 32.

Evaluation Metrics. In the field of gaze estimation, angular error is used as the primary metric to assess the accuracy of model performance. The angular error refers to the angular difference between the predicted gaze direction and the ground truth gaze direction, typically represented by cosine similarity, as shown in Equation (19):

(19) $$L_{angular}=\frac{g\cdot \hat{g}}{\left||g||\cdot ||\hat{g}|\right|}$$

where *g* is the ground-truth gaze vector and $\hat{g}$ is the predicted gaze vector.

### 4.3. Experiments on the Detectable Face Subset of Gaze360

To demonstrate the superiority of the RSP-MCGaze model, it was trained and evaluated on the detectable face subset, and its performance was compared with existing state-of-the-art models.

**Figure 4:**
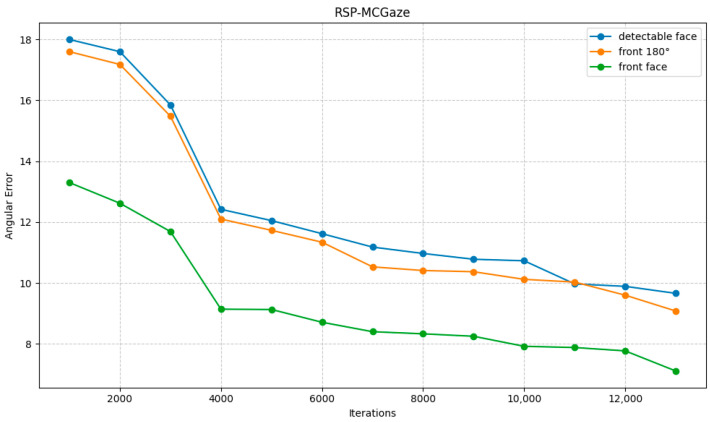
Performance Curves on the Detectable Face Dataset.

**Figure 5:**
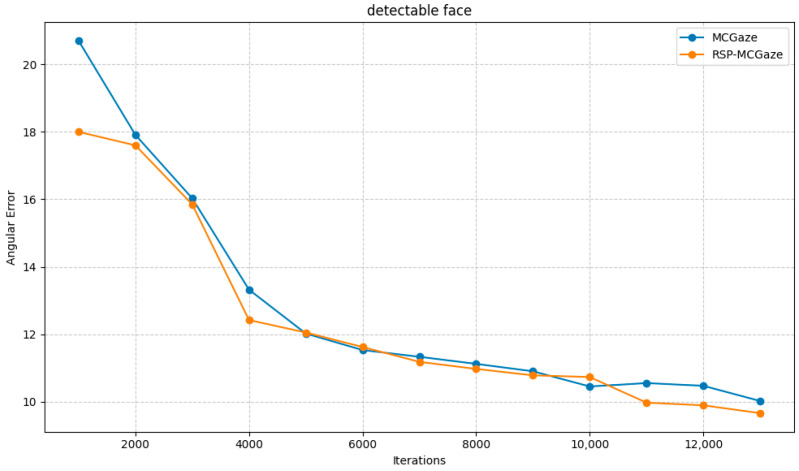
Detectable face.

**Figure 6:**
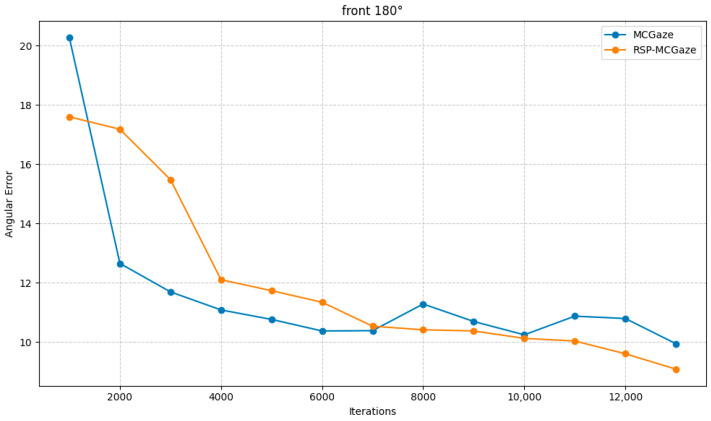
Front 180°.

**Figure 7:**
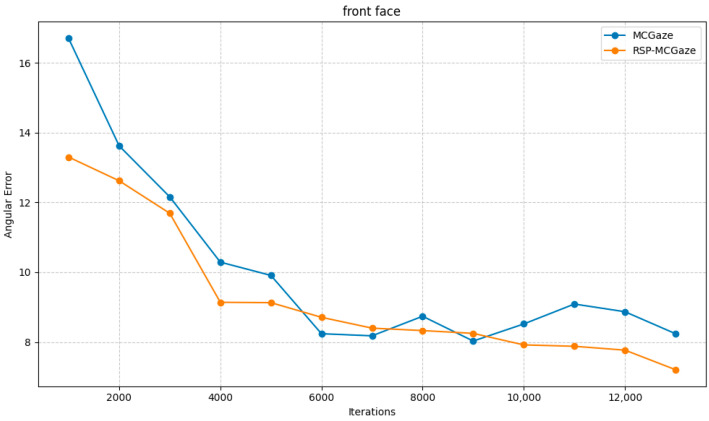
Front face.

Figure 4 shows the performance curve of RSP-MCGaze on the detectable face subset of Gaze360. Figure 5, Figure 6 and Figure 7 illustrate the comparison curves between RSP-MCGaze and MCGaze for the detectable face, front 180°, and front face categories on the detectable face subset of Gaze360, respectively. As seen in Figure 5, Figure 6 and Figure 7, RSP-MCGaze converges faster and achieves the best performance.

### 4.4. Experiments on the Entire Gaze360 Dataset

**Figure 8:**
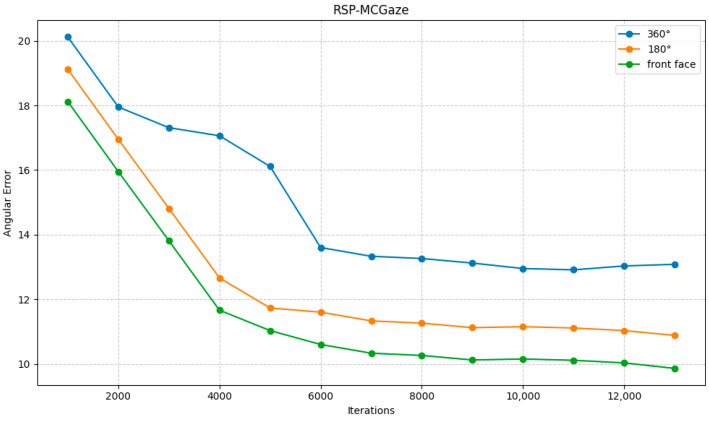
Performance curve on the Gaze360 dataset.

The RSP-MCGaze model was also trained and tested on the entire Gaze360 dataset to evaluate its generalizability. The performance curve is shown in Figure 8.

**Figure 9:**
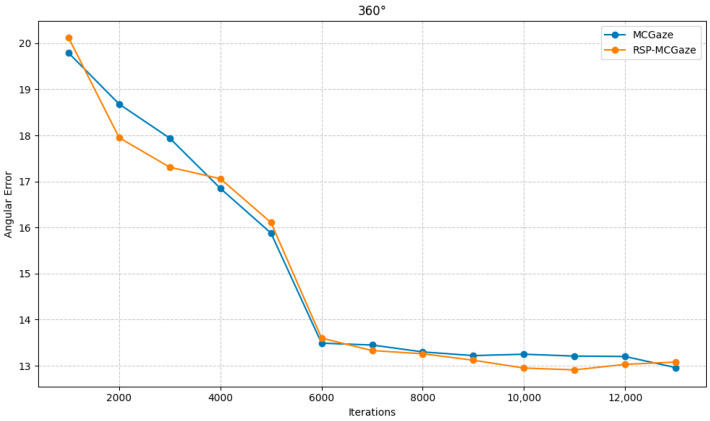
360°.

**Figure 10:**
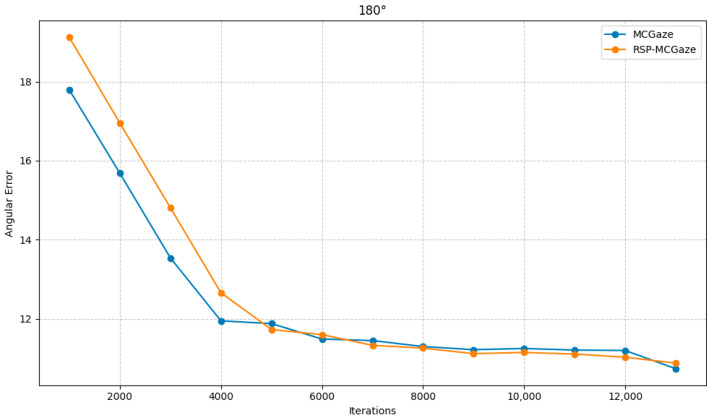
180°.

**Figure 11:**
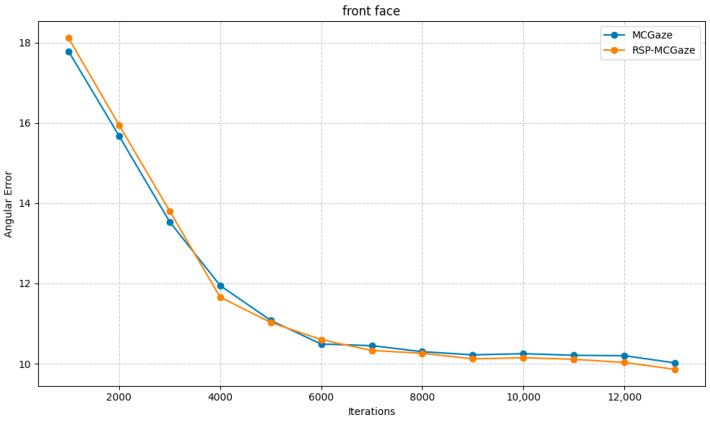
Front face.

RSP-MCGaze was also compared with the MCGaze model on the Gaze360 dataset in three settings: 360°, 180°, and front face. As shown in Figure 9, Figure 10 and Figure 11, RSP-MCGaze outperforms MCGaze in overall performance.

### 4.5. Experimental Results Analysis

The performance metrics of the proposed RSP-MCGaze model on the Gaze360 dataset and the detectable face subset, compared with other baseline models, are shown in Table 1 and Table 2, respectively:

(1) The proposed model, RSP-MCGaze, was tested on the detectable face subset of Gaze360 and achieved the lowest angular errors in all three metrics: 360°, 180°, and front face. This demonstrates the superiority of the RSP-MCGaze model.
(2) After comparing the RSP-MCGaze model with the MCGaze model, RSP-MCGaze achieved lower angular errors in the front face, 360°, and 180° key metrics. This demonstrates that the ResNet backbone, optimized by SCConv for feature extraction, can focus more on the important features of the head, face, and eyes through spatial and channel reconstruction. As a result, it achieves lower errors and superior performance in the gaze estimation task.
(3) Through experiments on the Gaze360 dataset using the RSP-MCGaze model, it was found that the model’s prediction performance is insufficient for parts of the data in the Gaze360 dataset where faces cannot be detected. The reason is that the RSP-MCGaze model is a gaze estimation model that focuses on the interrelationships between the head, face, and eyes. It requires clear images or videos of the face, head, and eyes to ensure the model’s gaze estimation performance.

## 5. Conclusions

Given the need to assess students’ learning concentration in online classrooms and considering that students’ gaze directions throughout the class can effectively reflect their level of attentiveness, gaze estimation can provide valuable insights for optimizing online education courses. Therefore, this paper proposes a gaze estimation model based on spatial-channel reconstruction and multi-cue localization (RSP-MCGaze). The RSP-MCGaze model, based on multi-cue optimization, further optimizes the backbone network by replacing the conventional 3 × 3 convolution with SCConv in the ResNet. This reduces redundant spatial-channel features and accurately extracts the important features that influence the gaze estimation task. Experiments demonstrate that the RSP-MCGaze model shows superiority and generalizability in the gaze estimation task under two settings of the Gaze360 dataset. It also confirms the model’s potential for application in online education, providing valuable theoretical references and technical support for the continuous optimization of online course design in the future.

## Figures and Tables

**Table 1:** Comparison of Angular Errors on Gaze360 Dataset.

Method	360°	180°	Front Face
Gaze360 [30]	13.50	11.40	11.10
MCGaze [6]	12.96	10.74	10.02
RSP-MCGaze (Ours)	13.03	10.88	9.86

**Table 2:** Comparison of Angular Errors on the Detectable Face Subset of Gaze360.

Method	Detectable Faces	Front 180°	Front Face
Gaze360 [30]	11.04	N/A	N/A
CA-Net [11]	11.20	N/A	N/A
GazeTR [26]	10.62	N/A	N/A
L2CS-Net [27]	10.60	10.41	9.04
SPMCCA-Net [28]	N/A	10.13	8.40
CADSE [12]	10.70	N/A	N/A
GazeNAS-ETH [29]	10.52	N/A	N/A
MCGaze [6]	10.02	9.81	7.57
RSP-MCGaze (Ours)	9.66	9.08	7.11

## Data Availability

The address of the public dataset Gaze360: http://gaze360.csail.mit.edu (accessed on 25 March 2025).
